# Cartilage-specific deletion of prar-gamma in mice results in early endochondral ossification defects and accelerated aging-dependent development of osteoarthritis

**DOI:** 10.1186/ar3632

**Published:** 2012-02-09

**Authors:** Roxana Monemdjou, Faezeh Vasheghani, Hassan Fahmi, Gemma Perez, Meryem Blati, Noboru Taniguchi, Martin Lotz, René St-Arnaud, Jean-Pierre Pelletier, Johanne Martel-Pelletier, Frank Beier, Mohit Kapoor

**Affiliations:** 1 Osteoarthritis Research Unit, University of Montreal Hospital Research Centre (CRCHUM) and Department of Medicine, University of Montreal, Montreal, Quebec, Canada, H2L 4M1; 2 Department of Molecular and Experimental Medicine, The Scripps Research Institute, La Jolla, California 92037, USA; 3 Genetics Unit, Shriners Hospital for Children, Montreal, Quebec, Canada, H3G 1A6; 4 Department of Physiology and Pharmacology, Schulich School of Medicine and Dentistry, University of Western Ontario, London, Ontario, Canada, N6A 5C1

## Background

Long bones develop through a strict coordinated process of endochondral ossification within the growth plate resulting in the replacement of cartilage by bone and defect in this coordinated process may result in skeletal abnormalities such as dwarfism, kyposis and also age-related defects such as osteoarthritis (OA). PPARγ, a transcription factor, plays a key role in lipid homeostasis but its in vivo role in cartilage/bone development is unknown. Therefore, we determined the specific *in vivo *role of PPARγ in endochondral bone ossification, cartilage/bone development and in OA using cartilage-specific PPARγ knockout (KO) mice.

## Materials and methods

Cartilage-specific PPARγ KO mice were generated using LoxP/Cre system. Histomorphometric/immunohistochemical analysis was performed to account for ossification patterns, chondrocyte proliferation, differentiation, hypertrophy, skeletal organization, bone density, calcium deposition and mouse OA phenotypic changes during aging using OARSI scoring. Real-Time PCR and western blotting was performed to determine the expression of key markers involved in endochondral ossification and cartilage degradation.

## Results

Histomorphometric analyses of embryonic and adult mutant mice demonstrate reduced long bone growth, calcium deposition, bone density, vascularity as well as delayed primary and secondary ossification. Mutant growth plates are disorganized with reduced cellularity, proliferation, differentiation, hypertrophy and loss of columnar organization. Isolated chondrocytes and cartilage explants from E16.5 and 3 weeks old mutant mice further show decreased expression of ECM production products, aggrecan and collagen II, and increased expression of catabolic enzyme, MMP-13. Furthermore, aged mutant mice exhibit accelerated OA-like phenotypes associated with enhanced cartilage degradation, synovial inflammation, and increased expression of MMP-13, and MMP-generated aggrecan and collagen II neoepitopes. Subsequently, we show that loss of PPARγ and subsequent downstream alterations in phosphatase and tensin homolog on chromosome ten (PTEN)/Akt pathway contribute towards increased expression of OA catabolic and inflammatory markers, thus enabling the articular cartilage of PPARγ-deficient mice to be more susceptible to degradation during aging.

## Conclusions

For the first time, we demonstrate that loss of PPARγ in the cartilage results in endochondral bone defects and subsequently accelerated OA in mice. PPARγ is essential for normal development of cartilage and bone.

